# Pharmacological treatments for patients with irritable bowel syndrome: An umbrella review of systematic reviews and meta-analyses

**DOI:** 10.1097/MD.0000000000015920

**Published:** 2019-08-09

**Authors:** Di Qin, Ling Yue, Bin Xue, Min Chen, Tai-Chun Tang, Hui Zheng

**Affiliations:** a The 3^rd^ Hospital/Acupuncture and Tuina School; b Acupuncture and Rehabilitation Department, Sichuan 2^nd^ Hospital of Traditional Chinese Medicine; c Clinical Medicine College/Teaching Hospital, Chengdu University of Traditional Chinese Medicine, China.

**Keywords:** irritable bowel syndrome, pharmacological treatments, protocol, study, systematic reviews

## Abstract

**Introduction:**

Several pharmacological treatments are beneficial for patients with irritable bowel syndrome (IBS), and there are numbers of systematic reviews evaluating the effectiveness of these treatments. However, the overall quality of the evidence has not been quantitatively assessed. The aim of this study is to evaluate the possible biases in the published systematic reviews and determine the treatments with reliable evidence.

**Methods and analysis:**

We will perform an umbrella review to identify eligible systematic reviews. A comprehensive literature search will be conducted in MEDLINE, EMBASE, and the Cochrane library for systematic reviews. We will describe the general information such as participants, interventions, outcome measurements, and conclusion. Additionally, the heterogeneity and inconsistency between trials will be assessed by the *I*^2^ statistical test and Cochrane *Q* test. We will assess risk of bias, and summarize the strength evidence.

**Conclusion:**

The umbrella reviews will assess the reliability of the evidence so that doctors and patients can make better medical choices.

**PROSPERO registration number:**

CRD42018109597

## 1 Introduction

Irritable bowel syndrome (IBS) is a chronic gastrointestinal disorder character by abdominal pain, discomfort, and altered bowel habit.^[1]^ Depending on the diagnostic criteria employed, IBS affects 9% to 23% of the population across the world.^[2]^

Internationally, there is a woman predominance in the prevalence of IBS. There is 25% less IBS diagnosed in those over 50 years and there is no association with socioeconomic status.^[3]^ IBS is a highly prevalent functional disorder that reduces patients’ quality of life and work productivity. It also has a large impact in direct healthcare costs.

IBS is subdivided to 3 subtypes irritable bowel syndrome with diarrhea (IBS-D), irritable bowel syndrome with constipation (IBS-C), or mixed irritable bowel syndrome (IBS-M).^[4]^ And visceral hypersensitivity is these subtypes common characteristic. The pathogenesis and pathophysiology of the visceral hypersensitivity of IBS remains incompletely understood. Dysregulation within the brain-gut axis and interactions between genetics, motor and sensory dysfunction, psychological factors, all likely play a role in the pathogenesis of IBS.^[5–7]^ The treatment focuses mainly on relieving symptoms and improving the quality of life.^[8]^ Pharmacological treatments are the primary choice because they are convenient and effective. Systematic reviews and guidelines recommended several pharmacological treatments, antispasmodic drugs are first considered to treat IBS. If patients are type of IBS-C, we consider offering laxatives to treat, but discourage use of lactulose. If patients are type of IBS-D, we choose loperamide as the first choice.^[9]^ However antispasmodic agents have low quality evidence because of small simple size, no high-quality trials, and other reasons. So we will choose other pharmacologic medications such as tricyclic antidepressants (TCAs), and 5-hydroxytryptamine type-3 antagonists (ramosetron alosetron, lubiprostone, and linaclotide).^[10,11]^ Antidepressant agents have become a widespread treatment for patients with moderate to severe IBS owing to their effects on pain perception, mood, and motility.^[12,13]^ Lots of randomized controlled trials (RCTs) prove TCAs are effective, but based on the potential for adverse effects, although they have high quality of evidence, the recommendation was weak.^[14]^ The 5-HT3 antagonist, alosetron, was shown a better effect than placebo at relieving IBS global symptoms with a high level of evidence, the 5-HT3 antagonist can slow colonic and small bowel transit and decrease intestinal secretion and colonic tone.^[15]^ But it was withdrawn from the market due to complications (ischemic colitis).^[16]^ Eluxadoline, one of opioid receptor agonists, plays a key role in regulating gastrointestinal motility, secretion, and visceral sensation. In vitro, eluxadoline reduces contractility in intestinal tissue and inhibits neurogenically mediated secretion,^[17]^ can improve abdominal pain and stool consistency. All these pharmacological therapies available to treat IBS symptoms, but they are not uniformly effective,^[18]^ and many patients reported intolerance this drugs because of side effects. In addition to aforementioned treatments, probiotics and dietary supplements are often used to treat IBS. Probiotics are living bacteria which have health benefits for patients with IBS when administered in adequate amount. They modulate gut microbiome and thus correct dysbiosis of the microbiota environment to treat IBS. Today, commonly used probiotics are Gram-positive species such as the Lactobacillus and Bifidobacterium.^[19,20]^ Although probiotics have been demonstrated the potential use in improving gut microbiome,^[21–23]^ but the longer-term impact and safety of repeated use of probiotics on the gut microbiota remains unclear, high-quality evidence of treatments remain scarce and insufficient to guide clinical use.^[19]^ According to the food and drug administration (FDA), dietary supplements include vitamins, minerals, herbs, amino acids, and enzymes. And now, recognized dietary supplements include vitamin D, dietary fiber (e.g., methylcellulose) and so on. Nonetheless, due to lack large amounts of research, the long-term effects remained to be elucidated.^[24]^ The evidence for various therapies shows some differences across conditions.

Therefore, we will conduct an umbrella review and meta-analysis to answer the question: Are current systematic reviews examining the effectiveness of pharmacological treatments in treating IBS under the risk of high between-study heterogeneity, small-study effect, or excess of significance bias? We will assess the credibility of published systematic reviews in the effectiveness of pharmacological treatments for IBS, to help clinicians and patients to choose suitable treatment options.

## 2 Methods and analysis

### 2.1 Study design

Considering the large number of therapies for IBS and the availability of numerous systematic reviews that examined the efficacy of these interventions, we will conduct an umbrella review to assess the credibility of published systematic reviews in the efficacy or effectiveness of pharmacological treatments for IBS. We will include systematic reviews with quantitative synthesis of data from RCTs and exclude systematic reviews including RCTs <10 trials. But if systematic reviews didn’t conduct quantitative synthesis of data from RCTs, we will describe the results of systematic reviews in detail. We will include systematic reviews comparing pharmacological treatments with placebo or usual care. We will exclude meta-analyses with missing 95% CI, and also exclude narrative reviews, letters, non-RCTs meta-analyses, and systematic reviews without quantitative synthesis, to ensure that the accuracy of the final results. The protocol of review conforms to the Preferred Reporting Items for Systematic Reviews and Meta-analyses Protocols (PRISMA-P),^[25]^ and this review has been registered at PROSPERO (ID: CRD42018109597).

### 2.2 Ethics and dissemination

The result of this umbrella systematic is to assess the credibility of published systematic reviews in the effectiveness of pharmacological treatments for IBS, to help clinicians and patients to choose suitable treatment options. This review does not require ethical approval and will be reported in a peer-reviewed journal.

### 2.3 Search strategy

We will search the electronic databases MEDLINE, EMBASE, and the Cochrane library for systematic reviews from inception to Dec 31, 2018, looking for systematic reviews examined the effectiveness of pharmacological treatments in treating IBS. We develop a comprehensive search strategy to find out systematic reviews that examined the effectiveness of the recommended interventions, adjusted to account for differences in indexing across databases. The search strategy sample is provided in Table 1. When multiple systematic reviews working on the same question are found, we will retain the one with the largest number of RCTs included. The search strategy will use keywords and medical subject headings (MeSH) in combination to search the systematic reviews that we need. MeSH and keywords contain “IBS,” “systematic review,” and synonymous words. And language restrictions will not be used in this review.

**Table 1:**
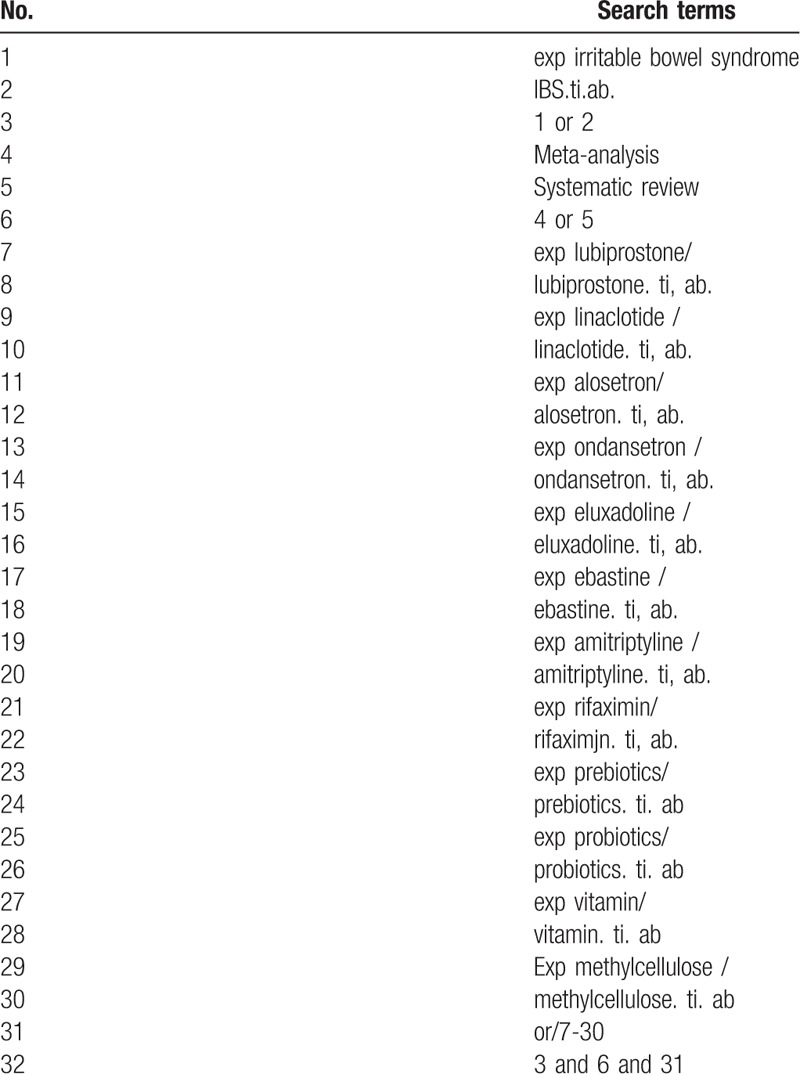
Search strategy.

### 2.4 Participants

Systematic reviews that include participants who are diagnosed with IBS by Rome I, II, III, or IV will be included. No restrictions will be set on IBS sub-types (constipation-dominated IBS, diarrhoea-dominated IBS, or mixed IBS), duration of IBS, or therapy types (monotherapy, dual therapy, triple therapy, etc.). We will exclude systematic reviews that include participants with inflammatory bowel diseases.

### 2.5 Interventions and comparisons

We will include systematic reviews that compare pharmacological treatments with placebo or other non-drug usual care. The non-drug usual care including acupuncture, diet therapy (e.g., low-FODMAP diet), exercise therapy, and psychological therapy. The pharmacological treatments will include: antispasmodics (dicyclomine, otilonium bromide pinaverium bromide),^[26]^ intestinal secretagogues (lubiprostone, linaclotide), 5-hydroxytryptamin type-3 receptor antagonist (alosetron, ondansetron), opioid receptor agonists (eluxadoline),^[27]^ antidepressants (TCAs, selective serotonin reuptake inhibitors [SSRIs]),^[28]^ probiotics (Lactobacilli, bifidobacteriae), and dietary supplements (vitamin D, diet fiber). The dose ranges of various drugs are shown in Table 2.^[29]^

**Table 2:**
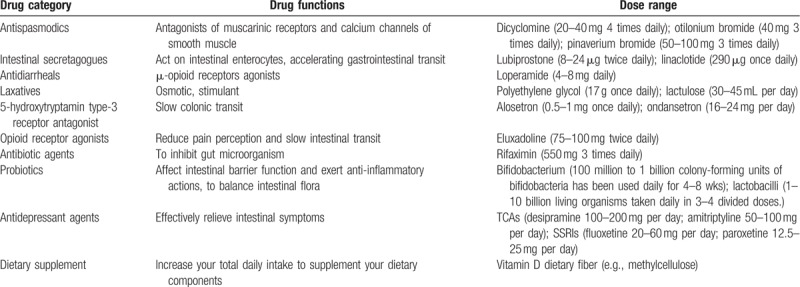
Drug category, function, and dose range.

### 2.6 Outcomes measurements

The primary outcome will be the improvement of global symptoms. Global symptoms include gastrointestinal symptoms like bloating, sensation of incomplete evacuation, straining (constipation), and urgency (diarrhea)^[30]^;and it also include psychological disorders like anxiety, depression, and other emotional disorders. The extent of the global symptoms will be assessed by using visual analog scale (VAS); it is a unidimensional measure providing a simple solution for measuring subjective experience. The VAS scale is a 10-cm line with 0 cm indicating no symptoms and 10 cm indicates the greatest extent of symptoms.^[31–33]^ If primary outcome is not defined in the systematic reviews, we will select outcomes that are suggested by FDA or outcomes that are used by most of the reviews in the recent 3 years.

The secondary outcomes will include: responder rate, improvement of major IBS symptoms (abdominal pain, bloating, and defecation urgency), stool consistency, stool frequency, and adverse events. The definition of responder will be determined according to each meta-analysis, and we limit the definition to significant improvement in global symptoms or abdominal pain according to the guidelines.^[9,34]^ Abdominal pain is a predominant feature of the IBS illness experience, which patients often feel pain cramping, stabbing, or sharp. Unlike other IBS symptoms, abdominal pain independently drives health related quality of life decrements in IBS.^[35]^ According to the standards defined by the FDA guideline,^[36]^ a responder refers to a patients who achieves at least 50% improvement in the extent of global symptoms or at least 30% improvement in the extent of abdominal pain.^[37]^ The intensity of abdominal pain could be measured by using VAS, numberic rating scale, or other Likert scales for pain measurement. Abnormal defecation can be evaluated by stool frequency and stool consistency. Stool frequency, measured by the number of complete spontaneous bowel movements (CSBMs) per week, <3 CSBMs per week considered abnormal.^[38]^ The Bristol Stool Form Scale, provides a pictorial and verbal description of stool consistency and form, it is an appropriate instrument for capturing stool consistency in IBS trials.^[39]^ It classify the form of human faeces into 7 categories: types 1 and 2 indicate constipation, with 4 being the ideal stools as they are easy to defecate while not containing excess liquid, 5 tending towards diarrhea, and 6 and 7 indicate diarrhea.^[40]^

### 2.7 Data extraction

After literature search, 2 reviewers (DQ and LY) will independently screen titles and abstracts of selected studies to remove duplicate papers and find out which are potentially eligible. If they cannot determine whether a systematic review should be included according to its title or abstract, they will further examine the full-text of the study. Another 2 reviewers (MC and TCT) will read the full-text and extract data from eligible studies with standardized sheets. First, we will extract the general information of the included name of first author, publication time, country, number of RCTs included in a meta-analysis, and the number of RCTs with positive results. Secondly, we will extract the information for a single meta-analysis, including the ID of a single meta-analysis, name of interventions and comparisons, and subtype of IBS. Finally, we will extract data from the reported outcomes. For continuous outcomes, we will extract mean, standardized deviation, and sample size; for category outcomes we will extract the number of events or responders and sample size.

### 2.8 Risk of bias assessment

The quality of eligible systematic reviews will be measured by A MeaSurement Tool to Assess systematic Reviews (AMSTAR), which is the most frequently mentioned tool for assessing the quality of SRs.^[41]^ AMSTAR consists of 11 items and has good content validity for measuring the methodological quality of systematic reviews.^[42]^ And it has good agreement, reliability, construct validity, and easy to use when assessing the quality of published SRs.^[43,44]^

We will assess the credibility of published systematic reviews in the effectiveness of pharmacological treatments for IBS. We will classify the credibility of the evidence into: convincing, suggestive, and weak evidence. Four criteria will be used to assess the credibility of current evidence: have *P* < .05 in fixed-effects model or *P* < .001 in random-effects model; have the total sample size >1000; have 95% prediction interval (PI) that excluded the null value; have small to moderate between-study heterogeneity (*I*^2^ <50%); and have no evidence of small-study effects or excess significance bias. Evidence meeting all the 4 criteria will be marked as convincing evidence; evidence meeting the first 3 criteria will be marked as suggestive evidence; and the others will be marked as weak evidence.

### 2.9 Data synthesis

This protocol will summarize the main findings of the eligible meta-analyses in systematic reviews. For systematic reviews without meta-analysis, we will perform descriptive analysis. For systematic reviews with meta-analysis, we will use random-effects model (meta package in R 3.5.0) calculate the summary ES and 95% CI, estimate the 95% prediction intervals (PIs). We will also assess whether they excluded null value. After we account for heterogeneity, the 95% PIs will provide ES information and its 95% CI in future trials. We will use *I*^2^ statistics to assess the between-study heterogeneity in each meta-analysis between-study. We will classify the heterogeneity as 3 degrees: small (*I*^2^ <25%), moderate (25% < *I*^2^ < 50%), and large (*I*^2^ >50%). And we will use the egger test to evaluate publication bias and small-study effect. For the purpose of evaluating the excessive significant bias, we are going to carry out a test to assess whether the observed number of studies (O) with significant results (positive studies with *P* < .05) is larger than their expected number.^[45]^ We will have estimated E for each meta-analysis as the sum of the statistical power for each RCT, which will be estimated by an algorithm adopting a non-central *t* distribution.^[46]^ The true ES for any meta-analysis is unknown, so we will use the ES of the largest study (with the smallest standard error) in a meta-analysis to substitute. Excess statistical significance for a single meta-analysis will be set at *P* < .1. The ratio of O versus E will also be calculated separately for each meta-analysis. All the statistical analyses will be done in R project (version 3.5.0, www.r-project.org).

### 2.10 Subgroup analysis

To address some potential problems we will perform a subgroup analysis. First, we will compare the result in meta-analyses with significant findings with meta-analyses without; second, we will compare the result in meta-analyses those showing large heterogeneity with those showing small heterogeneity; third, we will compare the result in meta-analyses having small-study effects with those not having. And if possible, we will compare meta-analyses recruiting adults with those recruiting children and adolescents.

## 3 Discussion

In recent years, there have been a large number of systematic reviews to assess the effectiveness of drug treatment IBS, but the reliability of these systematic reviews needs to be further studied due to different experimental designs and different levels of evidence. The umbrella reviews will assess the reliability of the evidence so that doctors and patients can make better medical choices. And if possible, we hope to stimulate the interest of public health decisions so that can help them make better decisions.

## Acknowledgments

The authors thank the staff from the library of Chengdu University of Traditional Chinese Medicine for suggestions in developing search strategy for this protocol.

## Author contributions

Di Qin, Ling Yue, Bin Xue, Min Chen, Tai-Chun Tang, and Hui Zheng contributed to the conception and design of the study protocol. The search strategy will be developed and run by Di Qin and Ling Yue, who will screen the title and abstract of the studies after running the search strategy. Min Chen and Tai-Chun Tang will screen full copies of the remaining studies after the title and abstract screening. Bin Xue develops the protocol of statistical analysis. Di Qin, Ling Yue, and Bin Xue wrote the first draft of the manuscript. All the authors revised the manuscript and approved it for publication.

**Conceptualization:** Di Qin, Ling Yue.

**Data curation:** Di Qin, Ling Yue, Min Chen, Tai-Chun Tang.

**Formal analysis:** Bin Xue, Hui Zheng.

**Funding acquisition:** Min Chen, Hui Zheng.

**Methodology:** Di Qin, Ling Yue, Min Chen, Tai-Chun Tang, Hui Zheng.

**Writing – original draft:** Di Qin, Ling Yue, Bin Xue.

**Writing – review & editing:** Di Qin, Ling Yue, Bin Xue, Min Chen, Tai-Chun Tang, Hui Zheng.

Hui Zheng orcid: 0000-0002-0494-1217.
